# Supplementary material for: A Novel Model for Arbitration Between Planning and Habitual Control Systems
Source: Front Neurorobot. 2019 Jul 11;13:52. doi: 10.3389/fnbot.2019.00052 (PMC6637733; doi:10.3389/fnbot.2019.00052)
Supplement: Supplementary file 1 [file Data_Sheet_1.PDF]

Supplementary Material

1 APPENDIX 1: ALGORITHMS

To move the arm, a motion function is used that is described in Algorithm 1. Algorithm APAC is also shown in Algorithm 2. This algorithm shows how APAC combines the habitual and planning through arbitration.

Algorithm 1 Motion function: function that calculates location of the simulated arm with respect to the applied angles to shoulder (α) and elbow (β). l_1 and l_2 are current length of forearm and arm while O indicates the origin of the plane, where the arm is attached.

set up an input vector including $(\alpha, \beta, l_1, l_2, O)$
$\alpha_1 = \alpha \times \frac{\pi}{180}$
$\beta_1 = \beta \times \frac{\pi}{180}$
$elbow_x = \cos(\alpha_1) \times l_1$
$elbow_y = \sin(\alpha_1) \times l_1$
$end_x = elbow_x + \cos(\beta_1 + \alpha_1) \times l_2$
$end_y = elbow_y + \sin(\beta_1 + \alpha_1) \times l_2$
return$([end_x + O[0], end_y + O[1]], [elbow_x + O[0], elbow_y + O[1]])$

2 APPENDIX 2: NETWORK ARCHITECTURE, PARAMETERS AND VARIABLES

It should be again noted that APAC and our proposed predictive actor-critic framework is the extension of DDPG. While preserving almost all parameters and features of the base DDPG model, APAC has added additional networks, which include the forward and inverse models.

The actor has two fully connected layers with *relu* activation function. These layers are connected to a fully connected output layer with the size of action dimension (here only two neurons). Each of neurons produces a continuous value for either the shoulder or the elbow. Since we need to learn to move the shoulder and elbow in both directions, we selected output activation function to be *tanh* which is multiplied by 180 degrees to obtain -180 to 180 degrees of movements. Note that each joint only moves between 0 to 180 degrees but it can move in two different directions. Actor learning rate is equal to 0.0001.

The critic has two hidden layers too. The first layer includes 400 fully connected neurons. In the second layer, the output of the actor (300 fully connected neurons) is combined with the output of the first layer (300 fully connected neurons) from the critic to build a fully connected layer with 600 neurons. The output of the critic is only one node since it will learn the value of taken action at each state. All activation functions selected to be *relu*. Learning rate is 0.001. Gamma value to update the TD rule is set to 0.99.

The forward model has three layers of 400, 300, and 4 fully connected neurons. Output layer of this network has 4 neurons to predict the future location of the robot arm (end-effector location and elbow location). All activation functions are *sigmoid*. The output of the network is multiplied by the dimension of the environment which is 30 centimetres to produce location of the end-effector and elbow in a Cartesian coordinate. Learning rate is set to 0.1, and the optimization function is ”Adamoptimizer” like all other networks.

Algorithm 2 Arbitrator Predictive Actor Critic

Randomly initialize critic network $Q(s, a|\theta^Q)$ and actor $\pi(s|\theta^\pi)$ with weights θ^Q and θ^π
Initialize target network Q' and π' with weights $\theta^{Q'} \leftarrow \theta^Q$, $\theta^{\pi'} \leftarrow \theta^\pi$
Randomly initialize forward learner network $fl(s, a|\theta^{f_F})$ and inverse learner network $f_I(s|\theta^{f_I})$ with weights θ^{f_L} and θ^{f_I}
Initialize target network Q' and π' with weights $\theta^{Q'} \leftarrow \theta^Q$, $\theta^{\pi'} \leftarrow \theta^\pi$
Initialize replay buffer R
for episode=1,M **do**
  Initialize a random process N for action exploration
  Receive initial observation state $s_1 = [X_t^{end}, X_t^{ellbow}, X^{target}]$
  for t=1,T **do**
    if episode<100 **then**
      Select action $a_t = f_I(s_t|\theta^{f_I}) + N_t$ by the inverse learner
    else
      Compute RPE
      if RPE<1 **then**
        Select action $a_t = \pi([X_t^{end}, X_t^{ellbow}, X^{target}]|\theta^\pi) + N_t$ by the actor
      else
        Select action $a_t = f_I([X_t^{end}, X_t^{ellbow}, X^{target}]|\theta^{f_I}) + N_t$ by the inverse model
      end if
    end if
    Feed a_t and current location of the arm to the forward model and observe predicted location of the arm $[X'^{end}_{t+1}, X'^{ellbow}_{t+1}]$
    Execute action a_t and observe reward r_t and observe new location of the arm $[XP_{t+1}^{end}, XP_{t+1}^{ellbow}]$
    Integrate predicted location from forward model and real location and build s_{t+1} along with target location
    Store transition (s_t, a_t, r_t, s_{t+1}) in R
    Sample a random minibatch of N transitions (s_i, a_i, r_i, s_{i+1}) from R
    Set $y_i = r_i + \gamma Q'(S_{i+1}, \pi'(s_{i+1}|\theta^{\pi'})|\theta^{Q'})$
    Update forward model by back propagating the error between predicted location and real location of the arm
    Update inverse model by back propagating the error between predicted action and real taken action by the arm
    Update critic by minimizing the loss: $L = 1/N \sum_i (y_i - Q(s_i, a_i|\theta^Q))^2$
    Update the actor using policy gradient method:
    $\nabla\theta^\pi J \approx 1/N \sum_i [\nabla_a Q(s, a|\theta^Q)|_{s=s_i, a=\pi(s_i)} \nabla_{\theta^\pi} \pi(s|\theta^\pi)|_{s=s_i}]$
    Update the inverse model by minimizing difference between action predicted by the inverse model to move from X_t to X_{t+1} with the actual action that transfered the plant from X_t to X_{t+1}
    Update the target networks:
    $\theta^{Q'} \leftarrow \tau\theta^Q + (1-\tau)\theta^{Q'}$
    $\theta^{\pi'} \leftarrow \tau\theta^\pi + (1-\tau)\theta^{\pi'}$
  end for
end for

The inverse model, similar to the actor, has three layers of 400, 300, and 2 fully connected neurons. Output layer of this network has 2 neurons to predict proper angles for elbow and shoulder. Activation functions for the first and the second layers are *relu*, and *tanh* is used at the output layer. The output of the network is multiplied by 180 degrees to obtain -180 to 180 degrees of movements similar to the actor.

An experience replay memory of size 1000 samples is used. Each time we train networks using a mini-batch of size 500. The smooth update parameter to update target networks is 0.001, and Adamoptimizer is

used as an optimization function for actor and critic networks. All the networks use 'L2' regularization with a weight decay by the amount of 0.001.

List of all parameters and variables of all three models (DDPG, SPAC and APAC) are listed here in tables S1 and S2.

Table S1. Common parameters for all networks and models

Parameter name	Value
Maximum number of trials	1000
Maximum number of steps in each trial	30
Discount factor (γ)	0.99
Soft target network update (τ)	0.001
Random seed for randomizing	None
Buffer size for experience replay memory	1000
Minibatch size	500
Size of a 2D planar space	30×30 cm
Origin of the arm fixed on 2D planar space	[15,15]
Initial length of forearm	8 cm
Initial length of arm	5 cm
Target zone	area with radius 0.5cm around target

Table S2. Network parameters and variables

Parameters	APAC
Input dimension to actor	6
Neurons in the first layer of actor	400
Activation func. in first layer of actor	relu
Neurons in the second layer of actor	300
Activation func. in second layer of actor	relu
Neurons in the output layer of actor	2
Scale of output of actor	180
Activation func. in output layer of actor	tanh
Actor optimization function	Adam optimizer
Actor learning rate	0.0001
Input dimension to critic	8
Neurons in the first layer of critic	400
Activation func. in first layer of critic	relu
Neurons in the second layer of critic	600
Activation func. in second layer of critic	relu
Neurons in the output layer of critic	1
Critic optimization function	Adam optimizer
Critic learning rate	0.001
Input dimension to forward model	6
Neurons in the first layer of forward model	400
Activation func. in first layer of forward model	sigmoid
Neurons in the second layer of forward model	300
Activation func. in second layer of forward model	sigmoid
Neurons in the output layer of forward model	4
Scale of output of forward model	30
Activation func. in output layer of forward model	sigmoid
Forward model optimization function	Adam optimizer
Forward model learning rate	0.01
Input dimension to inverse model	6
Neurons in the first layer of inverse model	400
Activation func. in first layer of inverse model	relu
Neurons in the second layer of inverse model	300
Activation func. in second layer of inverse model	relu
Neurons in the output layer of inverse model	2
Scale of output of inverse model	180
Activation func. in output layer of inverse model	tanh
Inverse model optimization function	Adam optimizer
Inverse model learning rate	0.01
